# Artificial intelligence in rheumatoid arthritis: current applications and future perspectives

**DOI:** 10.3389/fmed.2026.1914306

**Published:** 2026-08-26

**Authors:** Zhanhui Sun, Chengzhang Li, Kairui Zhu, Liying Xu, Yulong Mu, Guoyu Wang, Lu Shi, Youjie Li, Yanmei Li

**Affiliations:** 1 Second Clinical Medical College, Shandong Medical and Pharmaceutical University, Yantai, Shandong, China; 2 Department of Rheumatology and Immunology, Yantaishan Hospital Affiliated to Shandong Medical and Pharmaceutical University, Yantai, Shandong, China; 3 Department of Biochemistry and Molecular Biology, Shandong Medical and Pharmaceutical University, Yantai, Shandong, China

**Keywords:** artificial intelligence, clinical applications, deep learning, machine learning, rheumatoid arthritis

## Abstract

Rheumatoid arthritis (RA) is a highly prevalent systemic autoimmune disease characterized by a complex and partially understood pathogenesis. The substantial challenges in early identification and marked therapeutic heterogeneity pose a significant burden on affected patients. Despite notable advancements in diagnostic techniques and therapeutic interventions in recent years, optimal patient care remains hindered by several ongoing clinical challenges. To address these limitations, artificial intelligence (AI) has been increasingly integrated into the field of rheumatology. By leveraging its potent capabilities in large-scale data and medical image processing, AI offers diversified and individualized approaches to RA diagnosis and management. Herein, following a review of the fundamental concepts of RA and AI, we explore the multifaceted applications of AI across RA diagnosis, treatment response prediction, *de novo* drug discovery, and both clinical and basic science research. Furthermore, we provide a comprehensive analysis of the strengths and pitfalls of implementing these models in real-world clinical settings. By summarizing the AI technologies that either hold significant promise for practical clinical application or have already been translated into routine practice, this review aims to catalyze the progress of AI-guided precision medicine and drive the intelligent evolution of RA management.

## Introduction

Rheumatoid arthritis (RA) is a chronic, systemic autoimmune disease primarily characterized by erosive and symmetrical polyarthritis. With synovitis as its core pathological hallmark, RA predominantly afflicts movable joints, including those of the hands, feet, and wrists. Clinical manifestations predominantly comprise joint stiffness, pain, and swelling, frequently accompanied by a systemic inflammatory response. Furthermore, extra-articular involvements, such as pericarditis and pulmonary fibrosis, are commonly observed (1). As the disease progresses, patients with RA may suffer from joint deformity, loss of function, and ultimate disability, thereby imposing a substantial burden on both the affected individuals and society at large (2). The pathogenesis and development of RA are multifactorial, intertwining genetic predispositions with environmental triggers. Given the marked heterogeneity in disease progression among individuals, a definitive “gold standard” for clinical diagnosis remains elusive. Consequently, establishing a diagnosis typically necessitates a comprehensive clinical evaluation integrating imaging modalities, laboratory findings, and clinical data (3). Conventionally, the interpretation of such diagnostic data has relied heavily on the expertise of clinicians. However, the sheer volume and complexity of medical information render manual analysis susceptible to errors, which may lead to adverse clinical consequences. Therefore, there is an urgent need for advanced analytical tools to assist in the diagnosis and management of RA.

Artificial intelligence (AI) is a scientific discipline founded on the simulation, extension, and augmentation of human thought processes and behavior (4). Over the past decade, upgrades in computational hardware have driven substantial advancements in AI, particularly in the subdisciplines of machine learning (ML) and deep learning (DL) (5). As a subfield of AI, ML is an analytical approach that optimizes algorithms and models by learning from data. By integrating specific data and algorithms, ML empowers computational systems to autonomously generate decisions and predictions without explicit programming, a process that typically necessitates human supervision (6). DL, a further subset of ML, is designed to emulate the structural and functional intricacies of the human brain. By utilizing complex artificial neural networks, DL models are capable of autonomously processing tasks without explicit human oversight (7). Algorithms form the core of both ML and DL, defined as a finite sequence of well-defined instructions or operational steps aimed at solving a specific problem within a limited timeframe (8). ML models generally possess shallower architectures, commonly employing algorithms such as support vector machines (SVM), decision trees, and shallow neural networks, which are well-suited for processing small-scale datasets and relatively simple tasks (6). Compared to ML, DL typically utilizes higher-order architectures, including convolutional neural networks (CNNs), recurrent neural networks (RNNs), and artificial neural networks (ANNs). These advanced algorithms excel in handling complex, high-dimensional data, such as images, text, and sequences (9). Fundamentally, model construction involves learning from a known dataset (the training set), fine-tuning parameters on a similar database (the validation set), and ultimately evaluating performance on an independent database (the testing set). The feasibility and robustness of these models are subsequently assessed using standardized metrics, including accuracy, the area under the receiver operating characteristic curve (AUC-ROC), sensitivity, and specificity (10).

**Figure 1:**
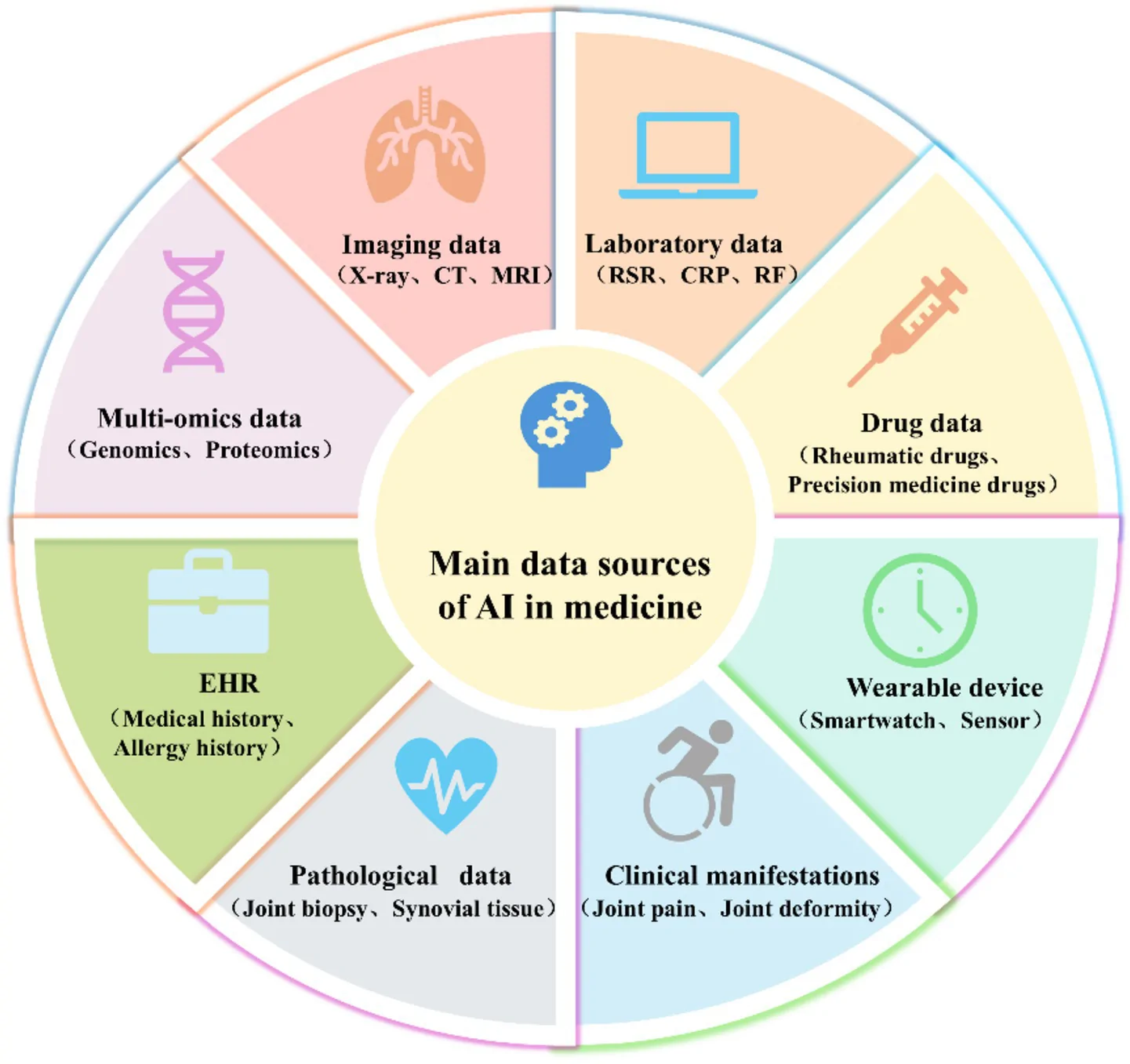
Core data sources of artificial intelligence.

Artificial intelligence (AI), leveraging its potent capabilities and computational speed in information processing, has made a significant mark on the medical field—spanning from early diagnosis to personalized therapy. To facilitate the management of RA, AI algorithms integrate a vast array of input data sources, including medical imaging, multi-omics data, clinical trial records, laboratory findings, electronic health records (EHRs), and data derived from wearable technologies (Figure 1). By mining these intricate datasets, AI can uncover the underlying mechanisms and intrinsic connections of the disease (11). Ultimately, AI—particularly deep learning—has been increasingly applied to the detection, diagnosis, and analysis of various conditions, including RA. These advanced computational models not only significantly enhance the efficiency and accuracy of disease diagnosis but also provide predictive insights to guide future therapeutic strategies.

In this review, artificial intelligence/machine learning (AI/ML) refers to data-driven approaches capable of learning decision rules, feature representations, clustering patterns, or sequential strategies from data. Conventional statistical methods, including ordinary linear regression, logistic regression, Cox proportional hazards regression, and regression-based nomograms, are not classified as artificial intelligence and are discussed only when they are used as comparator models or incorporated into hybrid modeling frameworks. Accordingly, performance evaluated on retrospective datasets or validation cohorts is described as technical performance or predictive performance. Claims of clinical utility or clinical effectiveness are made only when prospective studies demonstrate that an AI/ML-based approach has a measurable impact on clinical decision-making, diagnostic or therapeutic workflows, healthcare safety, or patient outcomes (12, 13).

**Figure 2:**
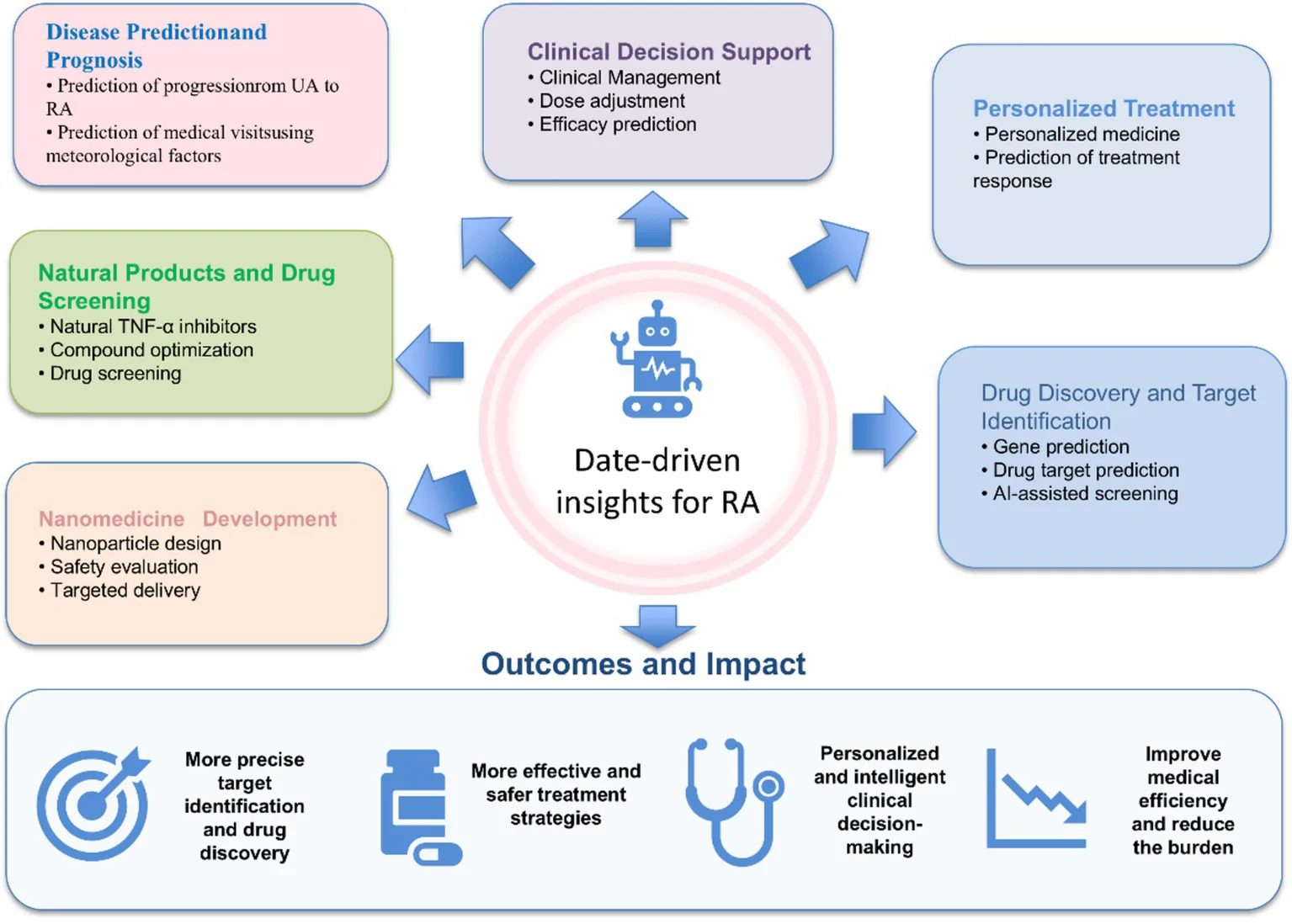
The applications and impacts of artificial intelligence in different fields.

Following an all-around disease-management framework, this review organizes the evidence according to each study’s primary clinical or translational purpose: diagnosis and classification, prediction of disease progression, personalized treatment and decision support, drug discovery and translational research, and wearable-assisted monitoring. Our primary objective is to underscore the rapid evolution of AI-driven tools in the context of RA care, delineate potential applications and future trajectories, and critically evaluate the performance of models with high translational potential (Figure 2).

## Diagnosis and classification

One of the major challenges in the diagnosis and management of rheumatoid arthritis (RA) is achieving timely and accurate disease identification. In the early stages of RA, inflammatory and structural abnormalities may be subtle, while clinical manifestations often overlap with those of other inflammatory and degenerative arthritides. To address these challenges, artificial intelligence/machine learning (AI/ML) approaches have increasingly been applied to integrate imaging, laboratory, clinical, and omics data for cross-sectional disease identification, lesion detection, severity grading, and differential diagnosis.

Radiography remains the most widely used imaging modality for evaluating RA-related bone erosions, periarticular osteoporosis, and joint space narrowing (JSN) (14). However, conventional image interpretation is time-consuming, subject to inter-reader variability, and relatively insensitive to subtle alterations in bone texture before overt erosive changes become apparent (15, 16). Kuo et al. developed a deep texture analysis model that integrated a curve graph convolutional network with a deep texture encoding network to identify RA-associated bone texture features on hand radiographs (15). Another study employed a lightweight VGG-8 convolutional neural network (CNN), together with data augmentation, gradient-weighted class activation mapping (Grad-CAM), and SHAP, and achieved favorable classification performance in the study dataset while enabling *post hoc* localization of regions such as the metacarpophalangeal and wrist joints (16). A customized CNN model, Custom3, achieved superior benchmark performance compared with the conventional machine-learning and pretrained models included in the same study (17). YOLOv5l6 has also been validated across different populations for automated localization of the proximal interphalangeal joints, metacarpophalangeal joints, wrists, radius, and ulna (18). The MediMatrix study further showed that grayscale pretraining yielded classification performance comparable to that achieved using pretraining on the color ImageNet dataset (19). Deep-TEN and ResNet-50 were also evaluated and were capable of identifying radiographic bone texture features that are difficult to interpret consistently by visual assessment alone (20). Overall, these studies demonstrate the technical feasibility of automated radiographic analysis; however, the performance of these models alone is insufficient to establish that they can optimize clinical diagnostic strategies or improve patient outcomes.

Unlike conventional radiography, infrared thermography enables noninvasive and radiation-free detection of inflammation-associated temperature gradients (21). One study evaluated machine-learning classifiers incorporating hand thermographic images for the identification of inflammatory arthritis (22), whereas another reported favorable classification performance for a hybrid RANet–support vector machine model constructed using multiview hand thermographic images (23). AI/ML approaches have also been explored in magnetic resonance imaging (MRI), ultrasonography, computed tomography, and nuclear medicine imaging (24). Automated segmentation and quantification models based on fuzzy C-means (FCM) clustering have been developed to segment and quantify synovial inflammation and bone marrow edema (BME) (25). Several studies have validated the ability of DenseNet models to identify metacarpophalangeal joint inflammation on two-dimensional grayscale ultrasound images (26). Neural network-assisted high-resolution peripheral quantitative computed tomography has also been used to quantify bone mineral density, bone microarchitecture, and bone morphology (27). Cobb et al. combined ^99mTc-maraciclatide imaging with an nnU-Net model and demonstrated segmentation reproducibility comparable to that of experienced radiologists (28). These findings support the technical performance of the respective imaging and analytical approaches; however, their impact on clinical diagnostic workflows remains to be established in prospective studies.

AI-based imaging technologies have evolved from general disease recognition toward quantitative assessment of individual pathological features. Validation across multiple datasets has shown that the ADMIRA framework can assess synovitis and BME with performance comparable to specialist interpretation (29). In musculoskeletal ultrasonography, high-resolution generative adversarial networks have been used to generate synthetic images of metacarpophalangeal synovitis, demonstrating the feasibility of image generation technology, although no direct diagnostic utility has yet been established (30). Computer-aided systems have also enabled automated or semi-automated bone localization and assessment of synovitis severity (31). For wrist MRI, a fully automated framework integrating super-resolution reconstruction, atlas-based segmentation, and fuzzy C-means clustering has been developed for volumetric quantification of BME (32). Deep learning-based radiomics models have also been evaluated for the detection of bone erosion on ultrasound images (33). An automated radiographic system incorporating YOLOv5, fine-tuned Segment Anything Model (SAM), Halo Edge Mask (HEM), and EfficientNetB0 has been developed to grade the severity of JSN (34). More comprehensive technical pipelines have integrated joint localization, bone contour segmentation, joint space width measurement, and bone erosion detection (35). Joint damage scores generated by the Mandora model have shown agreement with clinical assessments (36). These studies are included in this section because their primary endpoints concern cross-sectional lesion detection or grading. Most have demonstrated technical performance, but clinical effectiveness has not yet been established.

AI-assisted diagnosis is not limited to imaging. Conventional biomarkers, including rheumatoid factor and anti-cyclic citrullinated peptide antibodies, may yield false-positive or false-negative results and do not fully capture disease heterogeneity (37). Bai et al. developed an artificial neural network integrating age, sex, rheumatoid factor, anti-cyclic citrullinated peptide antibodies, 14-3-3η protein, and anti-carbamylated protein antibodies; in the study cohort, the model demonstrated greater discriminatory performance than any individual biomarker alone (38). Meng et al. combined ultrasensitive citrullinome profiling of minute samples with deep learning, enabling highly sensitive analysis of small-volume clinical specimens (39). A machine learning-optimized polygenic risk score identified nine key single-nucleotide polymorphisms and achieved an area under the curve greater than 0.97 in both the training and independent test datasets (40). Another study transformed clinical information, including complete blood count parameters, imaging findings, and joint symptoms, into two-dimensional array images and applied an AlexNet model for RA classification; the model showed a high level of agreement in the test set (41). These findings require further validation in independent prospective studies before such approaches can be regarded as clinically useful diagnostic tools.

AI/ML methods have also been investigated for RA phenotyping and differential diagnosis. Multimodal deep learning and clustering analyses based on the distribution and number of affected joints identified phenotypes characterized by predominant foot involvement, seropositive oligoarthritis, seronegative hand-predominant involvement, and polyarticular disease (42). Random forest analysis of serum protein biomarkers has been evaluated for distinguishing RA from psoriatic arthritis (PsA) (43). Another study used a three-dimensional residual network (ResNet) based on hand MRI to distinguish seropositive RA, seronegative RA, and PsA in the study dataset without requiring manual expert annotation (44). CNN and transfer-learning models based on hand radiographs have also been assessed for differentiating RA, osteoarthritis (OA), and healthy controls (45, 46). Deep learning systems have likewise been applied to identify calcium pyrophosphate deposition disease in existing OA and RA datasets (47). In addition, a study combining a plasmonic sensing platform with algorithmic analysis of synovial fluid Raman signals was able to differentiate RA from OA and stratify RA severity in the study samples (48).

Multimodal and spectroscopic approaches have further expanded the range of tools available for differential diagnosis. A TransCNN framework incorporating hyperspectral imaging was able to distinguish RA from clinically similar synovial lesions in the study dataset (49). Fourier-transform infrared spectroscopy combined with a multiscale convolutional network was used to differentiate RA from ankylosing spondylitis (50), whereas the ARAC-AOADL model was capable of classifying multiple osteoarticular disorders (51). Deep learning-based segmentation of MRI images of the suprapatellar bursa and infrapatellar fat pad has also been evaluated for differentiating RA, gouty arthritis, and pigmented villonodular synovitis (52). AI-based pathological image analysis may provide a foundation for future decision-support systems; however, such studies should report their datasets, reference standards, external validation procedures, and intended clinical applications in sufficient detail (53). Overall, the available evidence supports the technical feasibility of cross-sectional imaging recognition and differential diagnosis, whereas prospective evidence demonstrating an ability to improve clinical decision-making remains limited (Table 1).

**Table 1:** Brief overview of artificial intelligence in the diagnosis and classification of rheumatoid arthritis.

Main findings	Algorithms applied	Performance
Deep learning-based bone texture analysis differentiated RA from non-RA individuals and identified RA-associated radiographic texture alterations, suggesting potential utility for early diagnosis and monitoring of radiographic progression (15).	GCN, Deep-TEN, ResNet-18	AUROC = 0.68; texture score: RA = 0.49 (95% CI, 0.48–0.50) vs. non-RA = 0.42 (95% CI, 0.40–0.43), *p* < 0.01; texture score >0.43: adjusted OR = 3.42 (95% CI, 2.48–4.72).
VGG-8 differentiated RA, including early RA, from healthy controls on hand radiographs. Grad-CAM and SHAP localized model-relevant features primarily to clinically relevant regions, including the wrist and metacarpophalangeal joints and areas of joint-space narrowing and bone erosion (16).	VGG-8 CNN; Grad-CAM and SHAP for model interpretability	Training: AUC = 0.99, accuracy = 0.94, sensitivity = 0.95, specificity = 0.94, precision = 0.93; test: AUC = 0.81, accuracy = 0.74, sensitivity = 0.60, specificity = 0.88, precision = 0.83.
A customized patch-based CNN differentiated RA from normal hand radiographs; Custom3 showed the highest performance among the evaluated pretrained, customized, and feature-fusion approaches (17).	ResNet, GoogLeNet, Custom1–3 CNNs	Custom3: accuracy = 0.95, precision = 0.95, recall = 0.95, F1 score = 0.95.
YOLOv5l6 detected key hand and wrist joints on RA radiographs across different hand orientations and bilateral images (18).	YOLOv5l6	Mean precision = 0.998, recall = 0.983, mAP@0.1 = 0.993; joint-specific F1 score = 0.987–0.994.
MediMatrix classified X-ray images into five disease-severity categories and outperformed the evaluated comparison models for arthritis damage classification (19).	MediMatrix with grayscale ImageNet pretraining, ShuffleAttention (SA), ResSA blocks, and DensePath	μF1 = 0.7941, balanced accuracy = 0.8059, AUC = 0.9136, Cohen’s *κ* = 0.6846; grayscale fine-tuning accuracy = 91.36% on the Kaggle dataset.
DL-derived periarticular bone-texture features differentiated early RA from non-RA; ResNet-50 slightly outperformed Deep-TEN (20).	Curve-GCN for metacarpal segmentation; Deep-TEN (ResNet-18 backbone) and ResNet-50 for RA classification	Deep-TEN: AUC = 0.69, sensitivity = 0.67, specificity = 0.62, PPV = 0.64; ResNet-50: AUC = 0.73, sensitivity = 0.68, specificity = 0.67, PPV = 0.67.
Hand thermal imaging combined with AI differentiated RA from healthy controls; the RANet + SVM hybrid achieved the highest classification performance (23).	RANet, QNN, RF, SVM	RANet + SVM: accuracy = 97%, precision = 95%, recall = 98%, F1 score = 96%.
An automated MRI-based method segmented and quantified inflammatory tissue in RA hands and detected pre- and post-treatment changes consistent with manual assessment (25).	FCM	Sensitivity = 97.71%; positive predictive rate = 83.35%.
DL-based ultrasound analysis classified synovial proliferation and differentiated healthy from diseased RA joint conditions, supporting automated screening (26).	DenseNet-121	SP-no vs. SP-yes: AUC = 0.886, accuracy = 82.1%, sensitivity = 70.0%, specificity = 94.3%; healthy vs. diseased: AUC = 0.916, accuracy = 80.4%, sensitivity = 90.8%, specificity = 70.0%.
HR-pQCT joint-shape features enabled automated differentiation among RA, PsA, and healthy controls, with disease-specific regions visualized on 3D bone surfaces (27).	CSAE, Grad-CAM	CSAE (bone-mask input): RA AUROC = 75.39%; overall F1 score = 57.86%, precision = 59.20%, recall = 58.60%.
nnU-Net segmented normal-, low-, and high-inflammation regions on 99mTc-maraciclatide hand/wrist images and outperformed thresholding for synovitis assessment (28).	nnU-Net	Modified Dice = 0.94/0.51/0.76 for normal/low/high inflammation (overall = 0.74); AUC = 0.96, sensitivity = 0.98, specificity = 0.80.
ADMIRA estimated synovitis, tenosynovitis, and BME severity from hand/forefoot MRI, with performance approaching expert assessment, particularly for synovitis and tenosynovitis (29).	ADMIRA, CNN, CAM	Test set: R/ICC ≈ 0.90 for synovitis/tenosynovitis and ≈ 0.80 for BME; validation set: R/ICC ≈ 0.80 and ≈ 0.70, respectively.
The AI-assisted MSUS system used HRGAN-based augmentation to address limited, imbalanced data and classified MCP synovitis with accuracy comparable to experienced ultrasonographers and higher consistency (30).	HRGAN, Faster R-CNN, ResNet	ResNet-based system: accuracy = 83.49%; classification consistency = 100%.
MEDUSA provided semi-automated/automated assessment of synovitis severity from finger-joint ultrasound by detecting bone, skin, joint, and synovitis regions, with the aim of improving objectivity and repeatability (31).	RF	RF was selected as the final bone/skin detector based on performance and computational cost; exact accuracy/AUC values were not reported, and overall performance remained below that of experienced clinicians.
An automated MRI framework quantified wrist BME in early arthritis using image fusion, carpal-bone segmentation, and intensity clustering, showing good agreement with visual BME scores (32).	SRR, ABS, FCM	BME-QM vs. visual BME score: Pearson *r* = 0.83, *p* < 0.001; ABS precision = 0.92–0.96.
An ultrasound-based DLR model identified bone erosion in RA and improved diagnostic performance for both junior and senior sonographers (33).	DLR, ResNet-50, LASSO, LR, SVM	DLR_LR: internal test AUC = 0.966, accuracy = 0.905, sensitivity = 0.855, specificity = 0.939, F1 score = 0.879; external test AUC = 0.932, accuracy = 0.855, sensitivity = 0.831, specificity = 0.888, F1 score = 0.868.
An automated X-ray pipeline segmented joints and classified JSN severity in RA; HEM-based preprocessing produced the highest performance for both PIP and MCP joints (34).	YOLOv5, SAM	HEM + EfficientNetB0: PIP—AUROC = 0.9954, sensitivity = 1.000, specificity = 0.9869, balanced accuracy = 0.9934; MCP—AUROC = 0.9579, sensitivity = 1.000, specificity = 0.8398, balanced accuracy = 0.9199.
A fully automated radiographic system quantified JSN and bone erosions in RA, enabling reproducible continuous assessment of structural joint damage (35).	ShapeLLM, ASM	JSW: CV = 2–7%, mean absolute error = 0.19–0.40 mm; erosion detection: AUC = 0.89 (class I = 0.88; class II = 0.92).
Mandora combined joint-space segmentation with bilateral/multijoint information to quantify JSN and bone erosion on RA radiographs, with JSN performance approaching human scoring (36).	CNN, ETR, SHAP	Mandora: JSN—hand *r* = 0.895, RMSE = 0.489; foot *r* = 0.863, RMSE = 0.516. Erosion—hand *r* = 0.732, RMSE = 0.646; foot *r* = 0.711, RMSE = 1.01.
An ANN integrating demographic and serological variables differentiated RA from non-RA controls and outperformed the previous threshold-based approach; anti-CCP was the strongest predictor (38).	ANN, LR, RF, KNN	ANN: AUC = 0.951, accuracy = 0.906, precision = 0.927, recall = 0.905, F1 score = 0.916.
Citrullinome profiles combined with clinical indicators predicted RA treatment response, while a BiGRU model identified RA-sera-reactive citrullinated peptides for potential autoantigen discovery (39).	KNN, BiGRU	KNN external validation: AUROC = 0.883 (MTX + LEF) and 0.794 (MTX + HCQ); BiGRU: ELISA-validated accuracy = 84.2% for RA-sera reactivity prediction.
A 9-SNP ML signature and derived ML-PRS differentiated RA from controls across three independent external datasets, supporting genetic risk prediction (40).	SVM, NB, XGBoost	XGBoost (9 SNPs), external validation: AUC = 0.991–0.994, sensitivity = 0.936–0.945, specificity = 0.963–0.966, F1 score = 0.918–0.923, accuracy = 0.949–0.954.
CNNs applied to TDA-transformed clinical data differentiated RA from non-RA with performance comparable to expert rheumatologists (41).	AlexNet, ResNet-18	AlexNet: accuracy = 98%, precision = 91%, recall = 100%; agreement with rheumatologists: κ = 0.79–0.87.
Multimodal EHR-based clustering identified four reproducible RA phenotypes (JIP-foot, JIP-oligo, JIP-hand, and JIP-poly) with distinct MTX response, remission rates, and synovial histologic characteristics (42).	MMAE, PhenoGraph, SHAP	Cluster stability >80% over 1,000 iterations; MTX failure differed across clusters (*p* < 0.001), as did remission rates (*p* = 0.007).
Serum proteomics combined with ML identified a protein biomarker panel differentiating PsA from RA; the MRM-based panel retained discriminatory ability in an independent cohort (43).	RF	RF + MRM: AUC = 0.85 in the independent evaluation cohort; verification cohort AUC = 0.79.
Hand MRI-based DL differentiated seropositive RA, seronegative RA, and PsA based on inflammatory patterns; adding clinical and demographic variables provided little additional improvement (44).	3D-ResNet	3D-ResNet: seropositive RA vs. PsA—AUROC = 0.75, accuracy = 68%, sensitivity = 80%, specificity = 54%; seronegative RA vs. PsA—AUROC = 0.74.
A DL model detected CPPD features on hand radiographs, supporting differential diagnosis in RA, particularly seronegative RA, and screening for concomitant CPPD (47).	EfficientNetB4, Grad-CAM	Combined TFCC + MCP2 + MCP3 model: AUROC = 0.86, AUPR = 0.77, sensitivity = 0.77, specificity = 0.80, precision = 0.67, F1 score = 0.72.
SERS-based synovial-fluid profiling combined with ML differentiated RA from OA and classified RA severity by WBC level; metabolite profiling further supported candidate biomarker identification (48).	SVM, RF, LR, KNN	SVM: RA vs. OA—AUC = 0.98, accuracy = 98.1%, sensitivity = 97.3%, specificity = 100%; RA severity—accuracy = 98.1%, sensitivity = 98.7%, specificity = 98.7%.
HSI combined with DL differentiated RA from knee synovitis by integrating spatial morphology and spectral information; TransCNN outperformed the evaluated conventional DL models (49).	TransCNN, ResNet-34, DenseNet	TransCNN: accuracy = 91%, precision = 89%, recall = 90%, F1 score = 89%.
FTIR serum spectroscopy combined with DL differentiated RA, AS, and healthy controls; MSCNN showed the highest generalization and classification performance among the evaluated models (50).	ANN, CNN, AlexNet, MSCNN	MSCNN: AUC = 0.99, accuracy = 93.15%, sensitivity = 92.87%, specificity = 96.53%, precision = 93.52%.
MRI-based DL segmented the SC and IPFP and differentiated RA from GA and PVNS, with diagnostic performance comparable to experienced radiologists (52).	ResNet-18, U-Net, SVM, DT, AdaBoost, XGBoost	DT (RA, external test): accuracy = 0.82, precision = 0.75, recall = 0.60, specificity = 0.92, Dice = 0.67, AUC = 0.76.

## Prediction of disease progression

The clinical course of rheumatoid arthritis (RA) is difficult to predict. Patients with similar baseline characteristics may differ substantially in their subsequent risks of disease relapse, structural damage, joint deformity, and systemic complications. Unlike the cross-sectional diagnostic models described above, progression prediction models use baseline data or repeated longitudinal measurements to estimate future clinical states, identify changes in disease status over time, and identify patients who may require intensified follow-up. Importantly, predictive accuracy and external validation results reflect model performance and clinical validity rather than clinical effectiveness; they do not, by themselves, demonstrate that model-guided management improves patient outcomes.

Among patients with seronegative undifferentiated arthritis, a feedforward neural network developed using routine clinical and laboratory variables was able to predict progression to RA and maintained good discriminatory performance in an external validation cohort (54). This application represents disease progression prediction rather than disease classification, as the study endpoint was the transition from undifferentiated arthritis to RA. External validation strengthens evidence for the model’s generalizability and clinical validity; however, prospective interventional studies are still required to determine whether use of the model can facilitate earlier initiation of appropriate treatment and ultimately improve patient outcomes.

Long-term disease management also requires estimation of the risk of relapse and future disease activity. A study based on real-world longitudinal data achieved short-term prediction of disease flare following dose reduction or treatment discontinuation (55). An adaptive network, AdaptiveNet, has also been applied to disease progression prediction and showed relatively stable performance among patients with autoantibody positivity and longer disease duration (56). Electronic health record-based deep learning models have been developed to predict disease status at subsequent clinical visits, with predictive performance validated across different healthcare systems (57). Collectively, these studies support the feasibility of longitudinal prediction. Models evaluated using external datasets also provide some evidence of generalizability; however, current evidence remains insufficient to demonstrate that algorithm-guided follow-up can reduce disease relapse or structural damage.

Longitudinal imaging provides an objective means of quantifying structural damage and inflammatory changes over time. The automated rheumatoid arthritis scoring algorithm (AuRA) can identify progression of joint damage, with follow-up assessments showing agreement with expert scoring (58). A convolutional neural network (CNN)-based ultrasound model was developed to automatically measure metacarpal head cartilage thickness and, under the study conditions, achieved measurement precision exceeding the repeatability of repeated measurements performed by the same physician (59). Deep learning systems have also been evaluated for the detection of atlantoaxial subluxation and other cervical spine abnormalities (60), while a ResNet-101 model has been used to assess the risk of craniocervical junction abnormalities on cervical magnetic resonance images (61). An optimized YOLOv4 framework enabled automated localization and classification of lesions relevant to the modified Sharp/van der Heijde score (62). In addition, a one-dimensional convolutional neural network based on time–signal intensity curves derived from dynamic contrast-enhanced magnetic resonance imaging was developed for automated segmentation and quantitative assessment of hand synovitis (63). These studies demonstrate the technical feasibility of automated measurement and longitudinal assessment; nevertheless, there remains insufficient evidence that such technologies can alter clinical monitoring strategies or prevent joint deformity.

Machine-learning approaches have also been explored for risk assessment of systemic complications. Models integrating routine clinical variables with carotid ultrasonographic findings have been developed for cardiovascular risk stratification (64), whereas deep learning-based quantitative computed tomography has been used to identify early pulmonary abnormalities in patients with RA (65). To date, these studies have primarily demonstrated technical and predictive performance. Prospective validation across diverse patient populations and healthcare settings is required to determine whether these approaches can ultimately improve cardiovascular or pulmonary outcomes (Table 2).

**Table 2:** Brief overview of artificial intelligence in predicting disease progression in rheumatoid arthritis.

Main findings	Algorithms applied	Performance
Predictive models using routine clinical and laboratory variables predicted progression from seronegative UA to RA; FNN showed the highest performance (54).	FNN, GBC, RF, AdaBoost	FNN: training AUC = 0.924, accuracy = 87.8%; external validation AUC = 0.777, sensitivity = 77.8%, specificity = 66.3%, PPV = 52.2%.
ML models using longitudinal clinical data predicted 3-month flare risk in RA patients receiving bDMARDs, potentially informing treatment-tapering decisions (55).	LR, RF, k-NN, NB	Stacking ensemble: AUROC = 0.802 (95% CI, 0.717–0.887) using the full RETRO variable set.
AdaptiveNet predicted future RA disease activity from longitudinal clinical data, with better performance in seropositive patients and those with longer disease duration (56).	RNN, RF, LR, SVM	AdaptiveNet: active-disease classification—accuracy = 75.6%, sensitivity = 84.2%, specificity = 61.5%; regression MSE = 0.90, approximately 7% improvement over conventional ML models.
A longitudinal GRU-based DL model predicted RA disease activity at the next clinical visit and retained moderate performance in a different hospital system (57).	GRU, MLP	GRU-based DL model: internal test AUROC = 0.91 (95% CI, 0.86–0.96); cross-hospital external test AUROC = 0.74 (95% CI, 0.65–0.83).
AuRA quantified radiographic joint damage on hand and foot X-rays and tracked longitudinal structural progression, outperforming the top RA2-DREAM models in external validation (58).	YOLOv3, GBM	AuRA, external validation: RMSE = 23.6, *R*^2^ = 0.91, CCC = 0.96; longitudinal change vs. expert scoring: *r* = 0.74, *p* < 0.001.
A DL model detected cervical landmarks on X-rays and quantified AAS using ADI and SAC, supporting objective assessment of cervical spine involvement in RA (60).	HRNet, CNN	HRNet: key-point detection = 99.5%; AAS diagnosis by ADI: sensitivity = 0.86, specificity = 0.57; by SAC: sensitivity = 0.97, specificity = 0.50.
A ResNet-101 model predicted longitudinal development of upper cervical deformities (pannus and/or facet-joint degeneration) from baseline sagittal T2-weighted MRI (61).	CNN, ResNet-101	Accuracy = 0.84, AUC = 0.82, sensitivity = 0.75, specificity = 0.87, PPV = 0.56, NPV = 0.92.
A DL-based DCE-MRI method segmented and quantified hand synovitis using pixel-level TIC analysis, showing strong agreement with manual expert delineation (63).	1D-CNN, DCC, SELU	1D-CNN: sensitivity = 85%, specificity = 98%, accuracy = 99%, precision = 84%, Dice = 0.73; synovitis volume vs. manual segmentation: *r* = 0.87 (pixel level) and *r* = 0.84 (patient level).
ML integrating conventional risk factors, laboratory biomarkers, and carotid ultrasound phenotypes detected CVD and estimated 3-year CVD risk in a cohort including RA, outperforming conventional cardiovascular risk scores (64).	RF, SVM-RBF, LDA	SVM-RBF/RF (Cluster 3 + SMOTE): current CVD—accuracy = 99.93%, AUC = 1.00; 3-year CVD—accuracy = 99.85%, AUC = 1.00.
QCT analysis showed a lower percentage of normal lung and greater interstitial abnormalities in early RA than in healthy controls; abnormalities correlated with higher disease activity and older age (65).	2D and 3D CNNs	Evaluated using multivariable linear regression and correlation analyses; QCT interstitial percentage was inversely correlated with FVC % predicted.

## Personalized treatment and clinical decision support

Rheumatoid arthritis (RA) is a heterogeneous disease, and a uniform therapeutic strategy cannot ensure disease remission in all patients. With the development of precision medicine, artificial intelligence (AI) is gradually reshaping the traditional “one-size-fits-all” treatment paradigm. By integrating multidimensional clinical data with machine-learning algorithms, these approaches have the potential to improve the quality of clinical decision-making (66). AI/machine-learning methods, together with conventional statistical-learning approaches included as comparators in some studies, have been investigated for predicting treatment response and supporting the selection of candidate drugs or dosing strategies (67). For biologic disease-modifying antirheumatic drugs (bDMARDs), one study compared two categories of models for predicting one-year remission (68): regularized statistical-learning methods, including lasso and ridge regression, and algorithms classified in this review as machine learning, including support vector machines, random forests, and extreme gradient boosting (XGBoost). SHAP values were used for *post hoc* interpretability analysis, identifying variables associated with model predictions, including erythrocyte sedimentation rate and hemoglobin concentration. Importantly, these comparative results reflect predictive model performance rather than the clinical efficacy of the drugs themselves. Methotrexate remains a first-line treatment for RA, although a substantial proportion of patients experience an inadequate response (69). Models integrating clinical variables with genomic single-nucleotide polymorphisms have therefore been evaluated for predicting response to methotrexate therapy (70). Another study used tender joint count, swollen joint count, and C-reactive protein as input variables to compare a conventional Cox proportional hazards model, a linear multitask regression model, and a random survival forest for estimating the timing of treatment escalation (71).

For tumor necrosis factor inhibitors, one study developed a predictive model integrating clinical variables, including serum creatinine, vitamin D levels, and swollen joint count, with interleukin-10 and microRNA miR-106a-5p; the model demonstrated predictive performance in an independent cohort (72). Researchers have also used synovial RNA-sequencing data from the STRAP clinical trial to establish molecular response signatures for etanercept, tocilizumab, and rituximab and subsequently translated these signatures into a rapid testing approach intended to support treatment selection (73). Independent validation increases confidence in the robustness and reproducibility of such biomarkers; however, prospective comparative studies are still required to determine whether biomarker-guided treatment selection leads to superior clinical outcomes compared with standard care.

AI/ML approaches have also been explored for coordinating and implementing personalized care. With the emergence of large language models such as ChatGPT, barriers to communication between patients and healthcare professionals may be reduced. Chen et al. suggested that such technologies may facilitate bidirectional communication by helping patients understand medical terminology and the potential benefits and risks of different treatment options, thereby supporting shared decision-making in clinical settings (74). A triage system based on natural language processing and machine learning was able to identify musculoskeletal disorders and patients requiring long-term care from unstructured clinical notes; within the test dataset, the system enabled automated risk stratification and prioritization for referral (75). Reinforcement-learning models have also been proposed to integrate digital biomarkers with medication data for sequential dose selection (76), while the multimodal MDD-CoD framework has been developed to match patient characteristics with candidate treatment options (77). At the healthcare-system level, long short-term memory models incorporating meteorological and air-pollution data have been used to forecast outpatient demand for RA care (78). Overall, these studies demonstrate the technical and operational feasibility of such approaches; however, evidence that they improve clinical outcomes, referral efficiency, or healthcare resource utilization remains limited (Table 3).

**Table 3:** Brief overview of artificial intelligence in personalized treatment and clinical decision support for rheumatoid arthritis.

Main findings	Algorithms applied	Performance
Regularized statistical-learning and ML models identified clinical features associated with remission after specific bDMARDs; variable importance differed by agent (68).	Lasso, ridge regression, SVM, RF, XGBoost	Across models: accuracy = 52.8–72.9%; AUROC = 0.511–0.694.
Adding 160 pharmacogenomic SNPs to clinical variables improved 3-month MTX response prediction in early RA compared with clinical data alone (70).	RF, SMOTE	RF (clinical + pharmacogenomics): training AUC = 0.84; external validation accuracy = 76.2%, sensitivity = 72%, specificity = 77%, PPV = 55%, NPV = 87%.
Predictive models estimated time to treatment adaptation in early RA; early treatment-response indicators outperformed baseline disease status (71).	Cox, LMT, RSF	Uno concordance index = 0.56–0.78 across baseline, week 4, and week 8 models.
Integrated clinical and molecular profiles identified patient phenotypes and signatures predictive of TNFi response in RA (72).	LR	AUC = 0.91.
Synovial-biopsy transcriptomics combined with ML predicted response to etanercept, tocilizumab, and rituximab; tocilizumab and rituximab models were externally validated (73).	EN, GBM, XGBoost, RF, SVM	EN (etanercept): AUC = 0.763; GBM (tocilizumab): AUC = 0.748, external AUC = 0.713; XGBoost (rituximab): AUC = 0.754, external AUC = 0.786; nCounter-converted models: AUC = 0.87, 0.82, and 0.87, respectively.
An ML pipeline using general-practitioner referral letters prioritized rheumatology referrals and identified RA, OA, and fibromyalgia cases (75).	XGBoost	RA: AUC-ROC = 0.78, AUC-PRC = 0.31; OA: AUC-ROC = 0.71, AUC-PRC = 0.44; fibromyalgia: AUC-ROC = 0.81, AUC-PRC = 0.33.
The Multimodal Data-Driven Chain-of-Decisions (MDD-CoD) framework integrated multimodal diagnostic and multi-attribute medication data for personalized medication decision-making in chronic diseases, including RA (77).	ViT, FT-Transformer, GNN	Outperformed baseline models across datasets; RA medication matching reached HIT@10 = 94%.
LSTM models forecast RA patient arrivals and outperformed traditional time-series models; meteorological and air-pollution variables further improved forecasting (78).	LSTM, ARIMA, SARIMA	Multivariable LSTM: test RMSE = 0.108, 0.119, and 0.109 for train:test splits of 2:1, 3:1, and 7:1, respectively.

## Drug discovery and translational research

Artificial intelligence (AI) can facilitate upstream research workflows by generating therapeutic hypotheses and prioritizing potential interventions. Its major applications include elucidating disease mechanisms, defining molecular phenotypes, identifying therapeutic targets, screening candidate compounds, and optimizing drug-delivery systems (79). The pathogenesis of rheumatoid arthritis (RA) involves complex interactions among genetic susceptibility, environmental exposures, immune dysregulation, activation of synovial stromal cells, and systemic inflammation (80). Machine-learning analysis identified formin-binding protein 1 as a candidate gene associated with air pollution; its expression was reduced in patients with RA and was correlated with disease activity, leading the investigators to propose a PM10–formin-binding protein 1–natural killer cell regulatory axis (81). Machine-learning analysis of gut microbiome profiles further identified bacterial genera potentially involved in immune regulation and inflammatory responses (82). Importantly, these computationally derived associations should be regarded as hypothesis-generating findings and do not establish causal relationships.

Unsupervised learning has also been applied to synovial RNA-sequencing data to identify an immune-inflammatory phenotype and a fibroblast-mediated tissue-destruction phenotype, suggesting that RA synovitis may comprise multiple distinct cellular regulatory programs (83). In another study, a support vector machine model integrating synovial histopathology, RNA-sequencing data, and clinical phenotypes classified disease into high-inflammatory, low-inflammatory, and mixed subtypes and identified distinct pain-regulatory mechanisms associated with each subtype (84). Such molecular stratification provides a foundation for subsequent subtype-oriented investigations; however, the clinical value of using these classifications to guide therapeutic selection remains to be established in prospective studies.

Multi-omics integration has further advanced mechanistic investigation and biomarker discovery (85). Joint analyses spanning genomic, transcriptomic, and proteomic data identified shared features between RA and ulcerative colitis, primarily involving immune activation, oxidative stress, and metabolic dysregulation (86). A multicriteria analytical framework based on deep learning and rough set theory was developed to address data conflict and nonparallel data structures in studies of disease progression and treatment heterogeneity (87). Another multimethod analytical pipeline combined the regularized regression method lasso with two machine-learning approaches, support vector machine recursive feature elimination and random forest, and identified RRM2, DLGAP5, and KIF11 as candidate genes from synovial gene-expression datasets, with the aim of identifying potential diagnostic and therapeutic targets for RA in synovial tissue (88). A separate analytical workflow integrated differential expression analysis, Gene Ontology/Kyoto Encyclopedia of Genes and Genomes enrichment analysis, protein–protein interaction networks, conventional logistic regression, and random forest algorithms to identify potential hub genes from peripheral blood messenger RNA profiles, with the aim of overcoming limitations of conventional serum biomarkers such as rheumatoid factor and anti-citrullinated protein antibodies in terms of sensitivity and early diagnosis (89). In this review, logistic regression is not classified as an AI method; only the random forest component is considered a machine-learning technique. These studies primarily generated candidate biomarkers, which require independent biological and clinical validation, and their diagnostic performance has not yet been established.

In targeted drug discovery, machine learning-based virtual screening is commonly used to prioritize candidate molecules (5). A CatBoost model incorporating molecular fingerprints identified potential JAK2 inhibitors (90). Graph convolutional networks were used to identify molecular features shared between RA and diffuse large B-cell lymphoma and to propose candidate drugs with potential cross-disease therapeutic relevance (91). An analytical framework integrating drug-induced expression profiles with gene-perturbation data was developed to predict potential drug targets and was evaluated using public databases (92). Deep learning-based virtual screening of natural products also identified candidate tumor necrosis factor-*α* inhibitors (93). Machine learning has additionally been used to model drug–nanocarrier interactions and assess nanomedicine safety. Computational models can prioritize drug–nanocarrier combinations for natural bioactive compounds with poor aqueous solubility and limited targeting capability (94). A machine-learning framework integrating Boruta feature selection, random forest-based toxicity prediction, and association rule mining systematically evaluated associations between nanomedicine toxicity and material properties, administered concentrations, and drug classes (95). Collectively, these findings demonstrate the feasibility of computational simulation and hypothesis generation. However, target-binding affinity, pharmacological activity, safety, and clinical efficacy of the proposed candidates require experimental and clinical validation (Table 4).

**Table 4:** Brief overview of artificial intelligence in drug discovery, wearable sensors, and remote monitoring for rheumatoid arthritis.

Main findings	Algorithms applied	Performance
Multimodal analysis and ML identified FNBP1 as an air-pollution-related RA gene; PM10 exposure was associated with impaired natural killer-cell cytotoxicity and exacerbated joint inflammation (81).	LR, NN-MLP, LDA, QDA, KNN, RF	RF: AUC > 0.75 across all three validation datasets; FNBP1 expression: AUC = 0.97, 0.78, and 0.95 in individual cohorts.
Gut microbiota and metabolite profiles were used to predict MTX response and classify RA disease stage and activity (82).	SVM-RFE, RF	Enterotype-based human index model for MTX response: training AUC = 0.945; microbial-based diagnostic models: AUC > 0.88.
Integrated synovial RNA-seq and histology identified three RA synovial subtypes (high-inflammatory, low-inflammatory, and mixed); histology-based ML predicted these molecular subtypes (84).	SVM, K-means	SVM: high-inflammatory vs. others AUC = 0.88; low-inflammatory vs. others AUC = 0.71; mixed vs. others AUC = 0.59; agreement with RNA-seq clustering = 39/45 (86.7%).
A multimodal, multicriteria conflict-analysis model combined DL with dominance-based rough sets for clinical nonparallel decision-making and was evaluated using an RA diagnosis dataset (87).	ResNet, DRSA, PCA	Reported classification accuracy up to 98.34% (CRC image classification) and mapped clinical parallel consistency/inconsistency distributions.
Gene co-expression analysis combined with ML identified a three-gene signature (RRM2, DLGAP5, KIF11) distinguishing RA from controls; a three-gene nomogram retained performance in an independent dataset (88).	WGCNA, LASSO, SVM-RFE, RF	Three-gene nomogram: training AUC = 0.935; external validation AUC = 0.908.
Blood transcriptomic analysis identified 52 differentially expressed genes and 9 hub genes and established a gene-based RA diagnostic signature (89).	LR, RF	High AUC values were reported across 5-fold cross-validation for both LR and RF; exact AUC values were not reported.
An ML workflow with experimental validation screened candidate small-molecule JAK2 inhibitors relevant to RA (90).	XGBoost, AdaBoost, LightGBM, CatBoost	CatBoost (test set): accuracy = 0.94, MCC = 0.86, AUROC = 0.98.
DL-based network analysis identified BCL2 and HSP90AA1 as molecular hubs shared by RA and DLBCL and prioritized them for drug-repurposing screening (91).	GCNConv, GNN	GCNConv: *R*^2^ = 0.9480/0.9288/0.9117 and RMSE = 0.0320/0.0448/0.0619 for training/validation/test sets, respectively; predicted scores = 0.766 for BCL2 and 0.714 for HSP90AA1.
ML using drug-induced and gene-perturbation expression profiles prioritized disease-specific drug targets, including PSMB8 and SMAD7 for RA, with literature support and external validation (92).	SVM, GBM, RF	Evaluated by ROC-AUC and PR-AUC; known drug targets from the OpenTargets database were significantly enriched across the evaluated diseases.
A DL-based virtual-screening model identified candidate natural TNF-α inhibitors, including Imperialine, Veratramine, and Gelsemine (93).	ANN	Test set: MSE = 0.6, MAPE = 10%, MAE = 0.5.
ML identified core-shell material, drug type/concentration, NP concentration, and cell type as major determinants of cytotoxicity in nanoparticle-based RA therapies; high drug concentrations and poly(aspartic acid)-based systems were associated with greater cytotoxicity (95).	RF, ARM	RF: RMSE = 6.6 (training), 18.1 (validation), and 16.0 (test).
A digital-health ML framework using smartphone and smartwatch data differentiated RA from healthy controls and estimated disease severity (97).	DCNN, SSL, HMM	RA vs. healthy controls: F1 score = 0.807; severity estimation with sensor-augmented patient-reported outcomes: F1 score = 0.833.
A wearable ISD combining tissue-impedance sensing with ML classified inflammatory status in an RA rat model, supporting real-time disease monitoring (98).	LR	LR: accuracy = 76.74%, AUC = 0.787.
A computer-vision model using standardized smartphone photographs detected joint inflammation in an Indian cohort and identified synovitis across specific hand joints (99).	CNN	MFPIP: accuracy = 83%, sensitivity = 77%, specificity = 88%; IFPIP: accuracy = 74%, sensitivity = 74%, specificity = 75%; wrist: accuracy = 62%, sensitivity = 90%, specificity = 21%.

## Wearable sensors and remote monitoring

Periodic outpatient follow-up provides only intermittent assessments of disease activity in rheumatoid arthritis (RA) and may fail to capture day-to-day fluctuations in pain, fatigue, physical function, and inflammatory status. Wearable sensors, smartphones, and connected devices enable continuous collection of real-world data. Wearable devices can record a range of physiological and behavioral parameters, including blood pressure, oxygen saturation, physical activity, joint mobility, and fatigue (96). Previous studies have analyzed smartphone- and wearable sensor-derived data to distinguish patients with RA from healthy individuals and to assess physical function (97). One study evaluated a reconfigurable integrated smart device combining conductive polymer microneedles with a machine learning-enabled electronic system for real-time detection of inflammatory status and investigated its use in conjunction with methotrexate under experimental conditions (98). Another study trained a convolutional neural network (CNN) using standardized smartphone images and evaluated its ability to detect synovitis of the wrist and proximal interphalangeal joints (99). Collectively, these studies demonstrate the feasibility of sensor-based monitoring and disease-related classification; however, it has not yet been established that home use of such devices improves disease control, medication adherence, or quality of life.

Technical accuracy alone is insufficient for successful translation of these technologies into clinical practice. Smuck et al. proposed seven key elements for integrating wearable devices into healthcare systems: defining the clinical problem, integrating the device into clinical workflows, providing technical support, personalizing the user experience, optimizing the user interface, establishing appropriate reimbursement mechanisms, and securing support from key clinical stakeholders (100). Future studies should establish standardized outcome measures, validate sensor-derived metrics against established clinical assessment methods, define alert thresholds and subsequent management pathways, and conduct prospective studies to determine whether remote monitoring can modify clinical management or improve disease outcomes.

## Potential challenges and mitigation strategies

Hallucination has emerged as an important safety concern associated with generative artificial intelligence and large language models (LLMs) in rheumatology (101). The use of these technologies raises several major concerns, including ethical issues, algorithmic bias, risks of plagiarism, limited originality, and the generation of hallucinatory content containing factual errors or inaccurate citations (102). In the field of rheumatoid arthritis (RA), language models have already been reported to generate incorrect or potentially misleading medical information (103). For example, Coskun et al. evaluated the performance of several LLMs in answering questions regarding methotrexate (MTX) use in RA. Although the models were generally able to provide accurate and comprehensive information, the study also identified instances of medical hallucination and misinformation in some models (104). Given the propensity of generative AI systems to produce plausible but misleading information, their use in clinical and patient-facing settings should therefore be approached with caution.

Artificial intelligence has advanced rapidly in biomedicine and has become increasingly integrated into medical diagnosis, disease monitoring, and personalized precision medicine. Nevertheless, substantial challenges and barriers continue to restrict its translation into real-world clinical practice. Data scarcity and poor data quality can directly impair model performance (105), while the lack of standardized data formats and definitions further hinders the development of robust and generalizable models (106). Algorithmic bias frequently arises from discrepancies between training datasets and the diversity of real-world clinical populations, potentially contributing to inequitable allocation of healthcare resources and inappropriate clinical decision-making (107). Many AI models also exhibit a “black-box” nature, with insufficient interpretability and transparency. Protection of patient data privacy, together with complex legal and ethical considerations, remains a central concern (108). Furthermore, variability in the quality and accuracy of training data, combined with the rapid evolution of AI technologies, creates uncertainty regarding whether current diagnostic standards and existing AI models will remain applicable over time (109).

**Figure 3:**
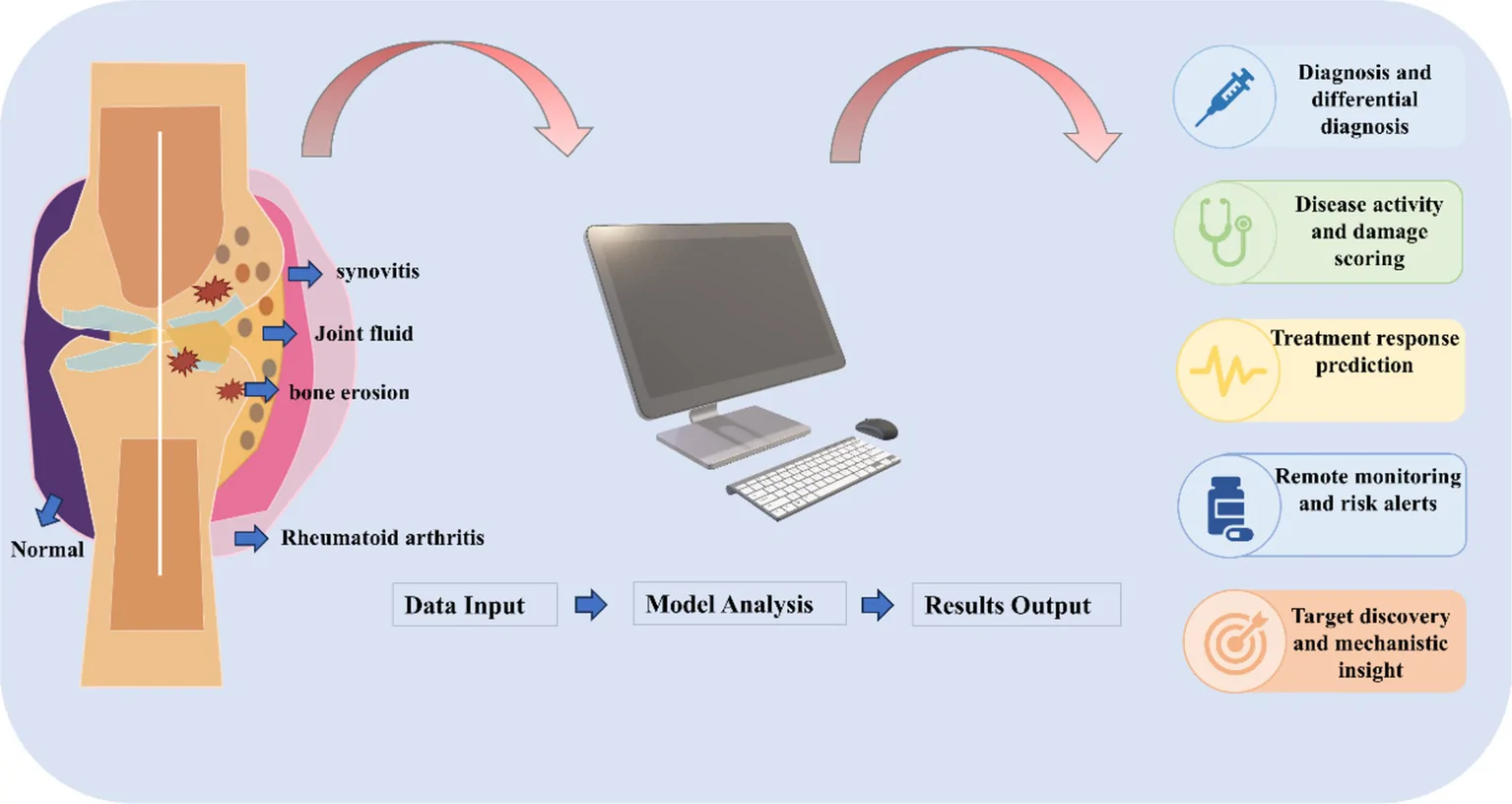
Overview of the analytical workflow from data input to model analysis and result output.

Addressing these limitations is therefore essential for the successful implementation of AI in real-world clinical practice (Figure 3). At the data level, efforts should focus on facilitating secure data sharing while improving the reliability, quality, interoperability, and standardization of healthcare datasets (106). High-quality training data constitute a fundamental prerequisite for the development of reliable medical AI systems. For example, when developing a detection model, Chen et al. adopted a rigorous reference-standard procedure involving independent image interpretation by multiple experts followed by consensus adjudication, thereby improving the reliability of the training dataset (110). Additional priorities include mitigating algorithmic bias and improving model interpretability to reduce the limitations associated with “black-box” decision-making, as well as establishing more standardized regulatory and approval pathways for AI-based medical products (111). Clinical implementation should also be supported by rigorous validation in prospective real-world studies and clinical trials. Closer interdisciplinary collaboration among clinicians, researchers, data scientists, and other relevant stakeholders will be essential for the responsible development and deployment of these technologies (112). Through continued optimization of data quality, model transparency, regulatory governance, and clinical validation, AI has the potential to contribute to more personalized, efficient, equitable, and standardized healthcare. However, the establishment of a fully digital, broadly accessible, and clinically reliable AI-enabled healthcare system will require sustained technical, regulatory, ethical, and clinical efforts.

## Conclusion

In summary, with the continued advancement of science and technology, an increasing number of medical devices and software applications are expected to incorporate artificial intelligence (AI). The integration of AI with diagnostic, monitoring, and therapeutic technologies has shown considerable potential to reshape the management of rheumatoid arthritis (RA), particularly by facilitating more precise disease assessment, longitudinal monitoring, and individualized treatment strategies. Such technological integration may provide new approaches for improving the detection, management, and therapeutic stratification of RA. Moreover, continued advances in AI may offer new opportunities for patients with refractory RA by supporting the development of targeted and personalized therapeutic strategies.

Future research should prioritize the development of robust, reliable, and generalizable algorithms and should incorporate external validation across diverse populations and healthcare settings to provide stronger evidence for model validity, transportability, and potential clinical utility. More importantly, prospective studies and real-world implementation studies are required to determine whether AI-assisted approaches can meaningfully improve clinical decision-making, healthcare delivery, and patient outcomes. With continued progress in methodological rigor, external validation, regulatory oversight, and clinical integration, AI has the potential to become an important component of future RA care and to contribute to more precise, efficient, and patient-centered healthcare.

## Data Availability

The original contributions presented in the study are included in the article/Supplementary material, further inquiries can be directed to the corresponding authors.
