# Supplementary material for: Artificial intelligence in rheumatoid arthritis: current applications and future perspectives
Source: Front Med (Lausanne). 2026 Aug 26;13:1914306. doi: 10.3389/fmed.2026.1914306 (PMC13553905; doi:10.3389/fmed.2026.1914306)
Supplement: Supplementary file 1 [file Table_1.DOCX]

| Stage of manuscript preparation | Generative AI tool | Purpose of use | Scope of use |
| --- | --- | --- | --- |
| Language revision and manuscript editing | DeepSeek | English-language polishing and grammatical correction | Used only to improve grammar, wording, sentence clarity, readability, and linguistic consistency. It was not used for manuscript restructuring, scientific content generation, literature retrieval or screening, data analysis or interpretation, conclusion formulation, or reference generation. |

**Supplementary Table. Disclosure of generative AI use during manuscript preparation**
